# Spray-Drying Microencapsulation of *Artemisia herba-alba* Phenolic Extract: Physicochemical Properties, Structural Characterization, and Bioactivity

**DOI:** 10.3390/molecules30193904

**Published:** 2025-09-27

**Authors:** Sara Lemmadi, Emilie Dumas, Faïza Adoui, Géraldine Agusti, Séverine Vessot-Crastes, Wafa Medfai, Adem Gharsallaoui

**Affiliations:** 1 Univ Lyon, Université Claude Bernard Lyon 1, LAGEPP, UMR 5007, 69622 Villeurbanne, France; sara.lemmadi@doc.umc.edu.dz (S.L.);; 2 Laboratoire de Génie Agro-Alimentaire (GENIAAL), Institut de la Nutrition, de l’Alimentation et des Technologies Agro-Alimentaires (I.N.A.T.A-A.), Université Frères Mentouri Constantine 1, Route de Ain El-Bey, Constantine 25000, Algeria; 3 Laboratoire de Biotechnologie de L’Olivier, Centre de Biotechnologie de Borj-Cedria, B.P. 901, Hammam-Lif 2050, Tunisia

**Keywords:** *Artemisia herba-alba*, phenolic compounds, microencapsulation, antioxidant activity, antibacterial activity

## Abstract

*Artemisia herba-alba* Asso. is a medicinal plant rich in phenolic compounds with strong antioxidant and antimicrobial activities. However, these bioactive molecules are highly sensitive to environmental conditions, limiting their stability and potential applications. This study investigated, for the first time, the encapsulation of ethanolic extracts from the aerial parts of *A. herba-alba* by spray-drying, using maltodextrin (MD) and sodium caseinate (SC) as wall materials. The extract was obtained by ultrasound-assisted extraction, and both free and encapsulated forms were analyzed for phytochemical composition, antioxidant capacity, and antibacterial activity. Spray-dried microcapsules (SDE) were further characterized for encapsulation yield, efficiency, moisture, water activity, hygroscopicity, particle size, and structural integrity (SEM, ATR-FTIR, TGA/DTG). The process resulted in a high encapsulation yield (69.40%) and efficiency (96.39%), producing microcapsules with a small average size (10.05 ± 0.08 µm), low moisture (4.34%), low water activity (0.415), and moderate hygroscopicity (12.67%). Although the encapsulated extract showed lower total phenolic content, antioxidant capacity, and antibacterial activity compared to the free extract, SEM observations confirmed the formation of spherical, crack-free microcapsules, ATR-FTIR analysis revealed non-covalent interactions between wall materials and phenolics, and TGA/DTG demonstrated improved thermal stability. These results highlight spray-drying microencapsulation as an efficient approach to stabilize *A. herba-alba* phenolic compounds, offering promising applications as natural preservatives in the food industry.

## 1. Introduction

*Artemisia herba-alba* Asso. is a silvery-green aromatic and medicinal plant [1,2] belonging to the genus *Artemisia* (Asteraceae), which includes more than 350 species [3]. It thrives in arid and semi-arid regions of the Mediterranean basin, North Africa, Spain, and the northwestern Himalayas [2,4,5,6,7]. Due to its wide distribution, it is known by different vernacular names, such as “Chih” in Arabic countries and “Armoise blanche” in France [8]. In traditional uses, it has been introduced into culinary practices—for instance, as a flavoring for tea in Tunisia [8] and for coffee in Algeria. In Algerian folk medicine, *A. herba-alba* is widely used to treat diabetes, hypertension, bronchitis [9], parasitic infections, coughs [10], colds [1], intestinal disorders, and neurological diseases [11]. Phytochemical studies have revealed that *A. herba-alba* is particularly rich in phenolic compounds, which confer a variety of biological activities, including antioxidant, antibacterial [12], and anti-inflammatory effects [10].

Over the past decades, growing interdisciplinary interest has been devoted to plant-derived extracts for applications in food [13,14], cosmetics, and pharmaceuticals [15]. This is largely due to their richness in phytochemicals, especially phenolics, well known for their potent antioxidant activity [14,16,17,18]. Their redox properties allow them to neutralize or scavenge free radicals, quench singlet or triplet oxygen, and decompose peroxides [19]. As safe and natural antioxidants [19], they are increasingly considered as substitutes for synthetic antioxidants, whose use is limited by potential toxicity and undesirable side effects [13,20]. However, the direct incorporation of phenolic compounds into formulations is hindered by their chemical instability and reactivity [21]. Their structures, often rich in conjugated double bonds, make them highly prone to oxidation, degradation, epimerization, and polymerization during processing and storage [22,23,24]. In addition, external factors such as oxygen [25], light [26], temperature [27], pH variation [28], humidity, metal ions, and enzymatic activities [22,29] can further compromise their bioactivity, leading to reduced bioavailability, decreased concentration, and shorter shelf life [30].

To overcome these limitations, encapsulation technologies have attracted increasing attention in sectors such as food [31], cosmetics [32], pharmaceuticals, textiles, chemicals, personal care, biotechnology, and medicine [21]. Encapsulation consists of entrapping bioactive molecules within protective matrices, forming particles at the macro-, micro-, or nanometric scale [33,34]. This strategy protects sensitive compounds from degradation [35,36], enhances their stability and bioactivity [32,35,37,38], and improves sensory quality by masking bitterness, astringency, or undesirable odors [39,40,41,42], while preventing color changes in the final products [43]. Various encapsulation techniques have been developed, including spray-drying [44,45,46,47], freeze-drying [42,48], inclusion complexation [49,50], coacervation [26,51,52], encapsulation in liposomes [53,54,55], nanoemulsions [17,40,56], supercritical fluids [57], and solvent evaporation [56,58]. Among these, spray-drying is considered the most industrially relevant, due to its profitability [59,60], simplicity [61,62], versatility, and low cost [28,63,64,65,66]. It also improves storage, transport, and instant solubility of the resulting powders [67,68]. Common spray-drying wall materials include polysaccharides (maltodextrins, starches, gum arabic, corn syrups), lipids (mono- and diglycerides, stearic acid), and proteins (gelatin, casein, whey, soy, wheat proteins) [29].

In this study, maltodextrin was selected as the primary encapsulating agent, consistent with previous findings highlighting its widespread use in spray drying [48,69]. Maltodextrin is non-toxic, renewable, biodegradable, and inexpensive [25]. It also exhibits favorable physicochemical properties such as neutral taste and odor [22,70], optical transparency [71], high solubility in water, and low viscosity even at high solids concentration [72,73]. Moreover, it provides thermal protection during drying and storage [67]. Nevertheless, its relatively low emulsifying power often necessitates combination with proteins, such as sodium caseinate, to improve interfacial properties and encapsulation efficiency [34,71,74,75]. Sodium caseinate, a salt derived from casein, is valued for its emulsifying and film-forming capacity, complementing polysaccharides that act primarily as fillers [60].

While several studies have characterized the biological activities of *A. herba-alba* phenolic compounds in different geographical contexts, their encapsulation has not yet been reported. Therefore, the present study focuses on the extraction of phenolic compounds from *A. herba-alba*, their encapsulation by spray-drying using maltodextrin and sodium caseinate as wall materials, and the subsequent evaluation of their antioxidant, antimicrobial, physicochemical, and structural properties.

## 2. Results and Discussion

### 2.1. Phenolic Content

**Figure 1:**
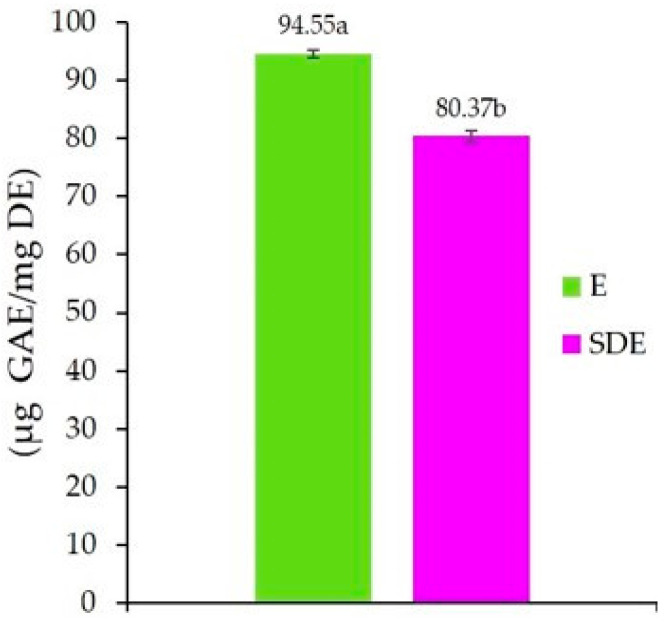
Total phenolic content (TPC) of the unencapsulated ethanolic extract of *A. herba-alba* (E) and the encapsulated extract obtained by spray-drying (SDE). The TPC is expressed as µg Gallic Acid Equivalent/mg of Dry Extract (µg GAE/mg DE). Data are expressed as the mean of three independent measurements (mean ± SD). Different letters (a, b) indicate significant differences between values at *p* < 0.05.

As shown in Figure 1, the total phenolic content (TPC) of the unencapsulated ethanolic extract of *A. herba-alba* (E) was 94.55 µg GAE/mg DE. This value is higher than that reported by Sekiou et al. [76] for an aqueous extract of *A. herba-alba* leaves (83.59 µg GAE/mg DE). Such differences can be attributed to several factors, including the part of the plant used, environmental and biological conditions influencing secondary metabolite biosynthesis [77], as well as external factors such as climate, soil composition, and geographical location [78]. In addition, the growth stage, harvest time [79], extraction method, storage conditions, and analytical techniques employed [79,80] can significantly affect the phenolic content of plant extracts.

Furthermore, as shown in Figure 1, a 15.00% reduction in phenolic content was observed after spray-drying, with the encapsulated extract (SDE) reaching 80.37 µg GAE/mg DE, compared to 94.55 µg GAE/mg DE for the unencapsulated extract (E). These findings are consistent with those of Kuck and Noreña [81], who reported phenolic compound losses ranging from 4.68% to 18.62% in *Vitis labrusca* var. Bordo grape skin extracts after spray-drying, when using partially hydrolyzed guar gum, gum Arabic, or polydextrose as encapsulating agents.

The observed decrease in phenolic content in SDE compared to the unencapsulated extract can be attributed to several degradation mechanisms: (i) mechanical degradation caused by the high shear force of the Ultra-Turrax homogenizer during feed solution preparation [82]; (ii) thermal degradation of heat-sensitive phenolic compounds due to the elevated inlet air temperature during spray-drying; (iii) oxidation reactions, triggered by exposure to oxygen and light throughout the drying process [81,83,84]; and (iv) interactions with wall materials, in which phenolic compounds become less extractable and less reactive to colorimetric assay reagents [85].

### 2.2. Antioxidant Activity

**Figure 2:**
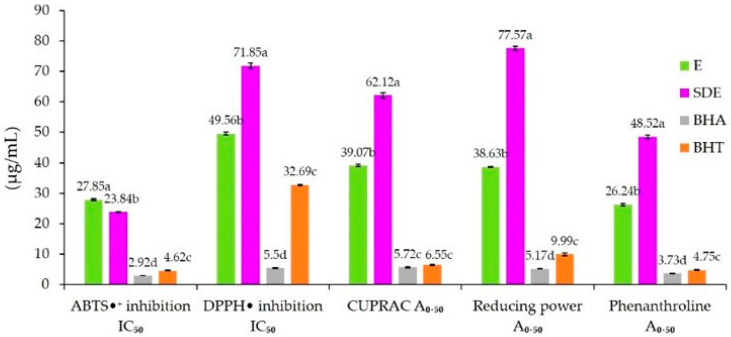
Antioxidant activity expressed as IC_50_ values (µg/mL) for ABTS•^+^ and DPPH• radical scavenging assays, and as A_0.50_ values (µg/mL) for CUPRAC, reducing power, and phenanthroline assays. Samples analyzed include the unencapsulated extract of *A. herba-alba* (E), the spray-dried microcapsule powder containing *A. herba-alba* extract (SDE), and the synthetic antioxidants BHA and BHT. Values are expressed as the mean ± SD of three independent replicates. For each assay, different letters (a, b, c, d) indicate significant differences among samples at *p* < 0.05.

The antioxidant activity of the ethanolic extract (E) and the spray-dried microcapsules (SDE) was evaluated using five complementary assays: ABTS•+ (2,2′-azino-bis(3-ethylbenzothiazoline-6-sulfonic acid) radical scavenging), DPPH• (2,2-diphenyl-1-picrylhydrazyl radical scavenging), CUPRAC (cupric ion reducing antioxidant capacity), reducing power (iron ion reduction), and the phenanthroline assay (Figure 2). The results showed that both extracts exhibited measurable antioxidant capacity, with differences between E and SDE depending on the assay applied.

These findings are consistent with the well-documented antioxidant role of phenolic compounds, which are among the most important groups of secondary plant metabolites [15,86,87]. Their antioxidant efficacy relies primarily on their redox properties, allowing them to act as reducing agents, hydrogen donors, oxygen quenchers, and metal chelators [88,89], and also to activate endogenous antioxidant enzymes [16]. However, as previously highlighted in the literature, the chemical diversity and complexity of phenolic-rich extracts make it difficult to rely on a single test for antioxidant evaluation. Therefore, combining multiple complementary assays is generally recommended to obtain a reliable and comprehensive assessment of antioxidant potential [90].

In this study, five complementary methods were used to evaluate the antioxidant activity of unencapsulated (E) and encapsulated (SDE) extracts: ABTS•^+^, DPPH•, CUPRAC, reducing power, and phenanthroline assays. The results are presented in Figure 2. For the unencapsulated extract (E), the antioxidant activity values were as follows: IC_50_ of 27.85 µg/mL (ABTS•^+^) and 49.56 µg/mL (DPPH•), and A_0.50_ values of 39.07 µg/mL (CUPRAC), 38.63 µg/mL (reducing power), and 26.24 µg/mL (phenanthroline).

Comparison with the literature highlights both similarities and discrepancies. The IC_50_ obtained with ABTS•^+^ was lower than that reported by Khlifi et al. [2] for a hydro-methanolic extract of *A. herba-alba* obtained by maceration (36.60 µg/mL). In the DPPH• assay, our IC_50_ was lower than the value reported by Dif et al. [91] (168.00 µg/mL), but higher than those reported by Bouchara et al. [92] (32.95 µg/mL) and Dhifallah et al. [1] (27.42 µg/mL) for ethanolic extracts of *A. herba-alba*. Regarding the reducing power assay, the A_0.50_ value obtained in this study (38.63 µg/mL) was considerably lower than that reported by Ayad et al. [93] (261.59 µg/mL), confirming the strong reducing ability of the extract analyzed in our work.

According to the data presented in Figure 2, the antioxidant activity of the encapsulated extract (SDE) was generally reduced compared to the unencapsulated extract (E). The IC_50_ and A_0.50_ values obtained for SDE were 23.84 µg/mL (ABTS•^+^), 71.85 µg/mL (DPPH•), 62.12 µg/mL (CUPRAC), 77.57 µg/mL (reducing power), and 48.52 µg/mL (phenanthroline). With the exception of the ABTS•^+^ assay, for which the IC_50_ of SDE was significantly lower than that of E, all other assays showed significantly higher IC_50_ or A_0.50_ values, indicating a reduction in antioxidant potential after encapsulation.

These findings are consistent with those of Ghandehari Yazdi et al. [94], Soleimanifar et al. [85], Ferreira et al. [95], and Akbarmehr et al. [96], who also reported decreased antioxidant capacity of phenolic extracts after spray-drying encapsulation. This reduction can be directly related to the decrease in total phenolic content (TPC) observed in the microcapsules (Section 2.1), which is itself attributed to various forms of phenolic degradation during homogenization and spray-drying processes.

### 2.3. Antibacterial Activity

The antibacterial activity of the unencapsulated ethanolic extract of *A. herba-alba* (E) and the encapsulated extract obtained by spray-drying (SDE) was evaluated using the agar well diffusion method against five bacterial strains (two Gram-positive and three Gram-negative). The diameters of the inhibition zones are summarized in Table 1.

According to the results, both E and SDE exhibited activity against *Brochothrix thermosphacta* (Gram-positive) and *Pseudomonas aeruginosa* (Gram-negative), while no inhibitory effect was observed against *Listeria innocua*, *Salmonella enterica*, or *Escherichia coli*. Specifically, the unencapsulated extract (E) showed moderate antibacterial activity, with inhibition zones of 11.56 mm against *B. thermosphacta* and 11.00 mm against *P. aeruginosa*.

As shown in Table 1, the inhibition zone of the unencapsulated extract (E) against *P. aeruginosa* was higher than that reported by Ayad et al. [93], who obtained an inhibition zone of 8.80 mm with an ethanolic extract of *A. herba-alba* (31.25 mg/mL), and slightly lower than that reported by Younsi et al. [7], who observed an inhibition zone of 11.50 mm with a methanolic extract (100 mg/mL).

In contrast, the antibacterial activity of the encapsulated extract (SDE) was slightly lower than that of the unencapsulated extract (E), with inhibition zones of 8.92 mm against *B. thermosphacta* and 10.59 mm against *P. aeruginosa*. This reduction in activity can be explained by the lower phenolic content in the encapsulated extract compared to the unencapsulated one, supporting the positive correlation between phenolic compound concentration and antibacterial effect, as also reported by Ghomari et al. [97].

When compared to the positive control, chloramphenicol, both E and SDE displayed significantly lower antibacterial activities against all tested strains, except *P. aeruginosa*, where inhibition zones were comparable. Chloramphenicol showed strong antibacterial activity against *L. innocua*, *B. thermosphacta*, *S. enterica*, and *E. coli*, with inhibition zones of 29.21 mm, 32.93 mm, 30.12 mm, and 27.78 mm, respectively, and moderate activity against *P. aeruginosa* (12.00 mm).

### 2.4. Physicochemical Characterizations of Powder

#### 2.4.1. Encapsulation Yield

As shown in Table 2, the spray-drying encapsulation yield of the ethanolic extract of *A. herba-alba* using maltodextrin DE 19 and sodium caseinate as wall materials was 69.40%. This value was significantly higher than that reported by Mainente et al. [31], who obtained a yield of 59.20% for the microencapsulation of phenolic extracts from *Tilia tomentosa* Moench flowers using maltodextrin DE 19 as the sole encapsulating agent. In contrast, it was only slightly higher than the yield obtained by Pudziuvelyte et al. [30], who reported 66.97% for the spray-drying encapsulation of ethanolic extracts of *Elsholtzia ciliata*, prepared with a mixture of four wall polymers (sodium caseinate, resistant maltodextrin, skim milk, and β-cyclodextrin).

Differences between our results and those reported in previous studies can be attributed to various physicochemical factors involved in both feed formulation and spray-drying conditions. Tsali and Goula [98] highlighted several key parameters influencing encapsulation yield. A higher wall material-to-core ratio generally improves yield, as the excess polymer provides a more effective coating for the extract, increasing the glass transition temperature of the droplets and reducing powder adhesion to the chamber walls. Furthermore, Navarro-Flores et al. [99] demonstrated that the chemical nature of the encapsulating polymer influences the kinematic viscosity of the feed solution, with higher viscosity being inversely correlated with encapsulation yield.

Moreover, Medfai et al. [100] reported that encapsulation yield is also influenced by several physical parameters, including the atomizer feed rate, equipment configuration, and particularly the inlet air temperature during spray-drying. A sufficiently high inlet temperature promotes rapid heat transfer, leading to faster solvent evaporation and the early formation of a dried crust on the surface of the droplets, thereby reducing the risk of particle adhesion to the walls of the drying chamber. However, an excessive increase in inlet temperature beyond the glass transition temperature of the wall materials may induce a transition from a glassy (powdery) to a rubbery (softened) state. This physical change favors particle stickiness, increasing adhesion of microcapsules to the chamber walls and ultimately causing substantial losses in encapsulation yield [98].

#### 2.4.2. Encapsulation Efficiency

Encapsulation efficiency (EE) is a critical parameter for evaluating both the performance of the encapsulation process [101] and the quality of the resulting microcapsules. EE represents the proportion of active compounds (phenolics) effectively entrapped within the microcapsule matrix [95].

As shown in Table 2, the EE of the spray-dried microcapsules was 96.39%. This value was higher than those reported by Rajapaksha and Shimizu [60] (81.56% for *Camellia sinensis* ethanolic extracts encapsulated with sodium caseinate and pectin) and Mainente et al. [31] (93.40% for *Tilia tomentosa* phenolics encapsulated with maltodextrin DE 19). However, it was slightly lower than the EE reported by Ferreira et al. [95] (96.60%) for *Astrocaryum vulgare* seed extracts encapsulated with maltodextrin DE 10.

Our findings are consistent with Cilek et al. [37], who emphasized that high EE values reflect the low proportion of phenolics located on the surface of microcapsules. The chemical composition of wall materials plays a decisive role in this parameter [83,102]. The combination of proteins and polysaccharides enhances the ability of phenolic compounds to interact with encapsulating agents, improving stability. Polysaccharides such as maltodextrin form the structural matrix, binding phenolics via non-covalent interactions (hydrogen bonds, hydrophobic interactions) [103]. Proteins like sodium caseinate provide emulsifying properties due to their amphiphilic nature, stabilizing phenolic compounds within microparticles [83]. Ultimately, the interaction depends on the structural characteristics of phenolics (molecular weight, hydroxyl group number, structural flexibility) and the concentration and type of wall materials used [104]. In line with Ghandehari Yazdi et al. [94], high wall-to-core ratios and increased mixture viscosity reduce phenolic migration to the surface, thereby improving EE.

#### 2.4.3. Moisture Content

Moisture corresponds to the amount of free and bound water in a food system [105]. The microcapsule powder obtained in this study (SDE) showed a moisture content of 4.34% (Table 2). This value, below the 6% threshold, ensures good stability and long-term preservation [95]. Moisture plays a key role in powder stability, influencing adhesion, flowability [106], oxidation of bioactives, and microbial growth [107].

Several factors affect moisture content, including feed rate [108], inlet air temperature [46,109], drying air flow rate (which impacts droplet size and evaporation rate [46]), and the chemical composition of the wall materials [69].

#### 2.4.4. Water Activity

Water activity (*a*_w_) of the spray-dried microcapsules was 0.415 (Table 2). This parameter reflects the amount of free water available in the system [42,110] and directly impacts microbial and biochemical stability of powders [111]. According to Mohammed et al. [6], powders with *a*_w_ values below 0.6 are considered microbiologically and enzymatically stable, supporting their suitability for long-term storage. As with moisture, water activity depends on factors such as wall material type and concentration, feed rate, and inlet temperature [102].

#### 2.4.5. Hygroscopicity

The hygroscopicity of SDE was 12.67% (Table 2). Hygroscopicity reflects the ability of powders to absorb moisture from the surrounding environment, which directly affects their flowability, physicochemical stability, and shelf life [96,112]. This property is influenced by both formulation and process parameters, particularly the chemical structure and concentration of wall polymers [69,113], as well as feed rate and drying air temperature [108].

#### 2.4.6. Particle Size

Particle size is a critical quality parameter affecting powder distribution, flowability, and retention of bioactive compounds [112]. It is determined by several factors, including wall material type and viscosity [114], feed dispersion homogenization rate, and spray-drying pressure [100].

The volume-weighted mean diameter (D [4,3]) of the microcapsules in this study was 10.05 ± 0.08 µm (Table 2). This fine particle size (<100 µm) is suitable for industrial food applications, as it avoids adverse effects on texture and sensory quality [95,115]. Larger particle sizes (>100 µm), however, may impart a granular mouthfeel when incorporated into foods, as noted by Joye and McClements [116] and Mehta et al. [24].

### 2.5. Structural Characterizations of Powder

#### 2.5.1. Morphology

Morphological analysis was performed by scanning electron microscopy (SEM) to investigate the shape and surface properties of the spray-dried microcapsules. SEM provided insights into the ability of the wall materials (maltodextrin, MD, and sodium caseinate, SC) to stabilize phenolic compounds within the microcapsule matrix and into the structural integrity of the formed barrier. The morphology of spray-dried microcapsules is generally influenced by the type, concentration, viscosity, and viscoelastic properties of the encapsulating agents [117].

**Figure 3:**
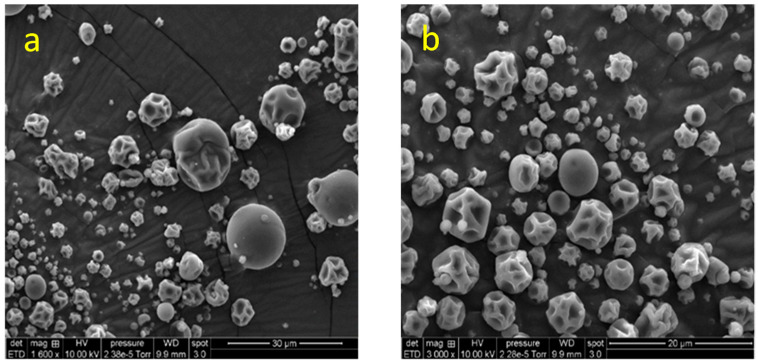
Scanning electron microscopy (SEM) images of spray-dried microcapsules containing *A. herba-alba* extract (SDE): (**a**) magnification ×1600; (**b**) magnification ×3000. The microcapsules appear mostly spherical, with heterogeneous sizes and surface features ranging from smooth to slightly wrinkled, without visible cracks or fractures.

As shown in Figure 3, the spray-dried microcapsules containing *A. herba-alba* extract (SDE) exhibited spherical shapes with heterogeneous particle sizes. Most microparticles displayed smooth outer surfaces without cracks or fractures, while some presented slightly wrinkled surfaces. Similar morphological features have been reported by Carra et al. [84] for grape skin phenolic extracts encapsulated with pectin and casein. Wrinkling and irregularities on the surface of spray-dried microcapsules are commonly associated with rapid moisture loss during the initial drying phase [69,118].

In addition, our results corroborate those of Carra et al. [84], who suggested that the absence of particle agglomeration can be attributed to the high negative surface charge of casein at pH 6.5, which promotes electrostatic repulsion and particle stability. Importantly, the absence of cracks or fissures in the microcapsule structure observed here is consistent with the low surface phenolic content and the high encapsulation efficiency measured (Section 2.4.2), indicating efficient protection of the phenolic compounds within the core.

#### 2.5.2. ATR-FTIR Analysis

**Figure 4:**
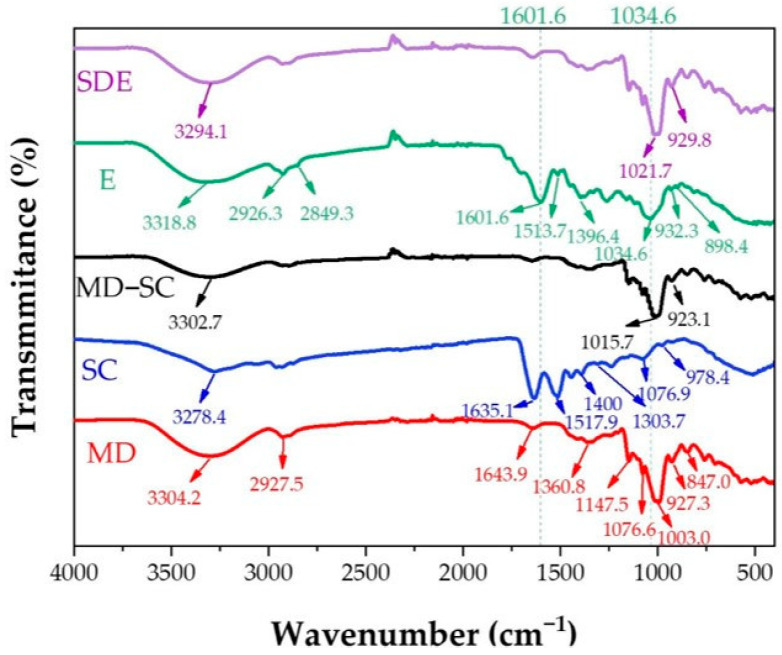
ATR-FTIR spectra of the unencapsulated ethanolic extract of *A. herba-alba* (E), maltodextrin powder (MD), sodium caseinate powder (SC), the MD–SC physical mixture, and the spray-dried encapsulated extract powder (SDE).

ATR-FTIR spectroscopy was carried out to identify the characteristic functional groups of the encapsulating materials and the core extract, and to assess possible interactions between maltodextrin (MD), sodium caseinate (SC), and the ethanolic extract (E). Figure 4 presents the ATR-FTIR spectra of the unencapsulated extract (E), MD powder, SC powder, the MD–SC physical mixture, and the spray-dried microcapsules containing the extract (SDE).

The spectrum of the unencapsulated extract (E) displayed several typical absorption bands. A broad band was observed at 3318.8 cm^−1^, generally attributed to –OH stretching vibrations of hydroxyl groups from residual water and phenolic compounds [94]. Peaks at 2926.3 cm^−1^ and 2849.3 cm^−1^ correspond to C–H_2_ and C–H_3_ stretching vibrations, typically associated with aromatic compounds containing phenyl groups such as flavonoids [119]. Absorptions were also detected at 1601.6, 1513.7, and 1396.4 cm^−1^, which can be assigned to C=C stretching vibrations in aromatic rings. A band at 1261.8 cm^−1^ is commonly attributed to C–O stretching of aromatic ethers, characteristic of flavonoid systems such as catechins [120]. Further peaks appeared at 1034.6 cm^−1^ (C–O–H bending [121]), 932.3 cm^−1^ (C–H bending [94]), and 898.4 cm^−1^ (C–C ring stretching [121]).

The spectrum of maltodextrin (MD) exhibited characteristic absorption bands at 3304.2 cm^−1^ (O–H stretching), 2927.5 cm^−1^ (C–H stretching of carboxylic groups), 1643.9 cm^−1^ (C=O stretching [6] and hydroxyl bending [44]), and 1360.8 cm^−1^ (–CH_2_, –CH, and =CH vibrations of carbohydrates [42]). Additional peaks were observed at 1147.5, 1076.6, and 1003.0 cm^−1^, corresponding to C–O stretching and C–O–H bending [6]. Peaks at 927.3 cm^−1^ (C–O–C stretching [122]) and 847.0 cm^−1^ (C–H vibrations [94]) were also detected.

The sodium caseinate (SC) spectrum exhibited peaks typical of proteins: 1635.1 cm^−1^ (amide I), 1517.9 cm^−1^ (amide II), and 1303.7 cm^−1^ (amide III), in agreement with previous studies [123,124]. Additional bands included 3278.4 cm^−1^ (–NH stretching), absorptions around 1400 cm^−1^ (carboxylate O–C–O groups), 1076.9 cm^−1^ (C–O stretching in C–OH groups), and 978.4 cm^−1^, attributed to monocationic interactions with Na^+^, also consistent with previous reports [124].

A comparison of spectra revealed notable shifts upon combination of the wall materials and the core extract. The O–H stretching band at 3304.2 cm^−1^ in MD shifted to 3302.7 cm^−1^ in the MD–SC mixture, and further to 3294.1 cm^−1^ in the SDE spectrum. This progressive shift indicates the formation of hydrogen bonds between the phenolic compounds and the polymeric matrix. Additionally, the characteristic peak of the extract at 2849.3 cm^−1^ disappeared in the SDE spectrum, suggesting chemical interactions between phenolics and wall materials, as previously observed by Akbarmehr et al. [96] with *Ilex paraguariensis* (yerba mate) extracts.

Further changes were observed in the carbohydrate-associated bands: in MD, the peaks at 1003.0 cm^−1^ and 927.3 cm^−1^ shifted to 1015.7 cm^−1^ and 923.1 cm^−1^ in the MD–SC spectrum, and to 1021.7 cm^−1^ and 929.8 cm^−1^ in SDE, accompanied by increased band intensity. According to Otálora et al. [125], such shifts are characteristic of non-covalent interactions, particularly hydrogen bonding between polysaccharides and phenolic compounds.

Moreover, several extract-specific bands between 1034.6 and 1601.6 cm^−1^ (E spectrum) were masked in the SDE spectrum by overlapping maltodextrin signals, indicating efficient incorporation of phenolic compounds into the encapsulating matrix. This agrees with the findings of Xue et al. [126], who demonstrated that wall materials can effectively cover the spectral region (1029–1692 cm^−1^), confirming integration of core and coating materials.

Overall, these results support the observations of Rigolon et al. [127], who also reported the presence of hydrogen bonding in encapsulated systems. The ATR-FTIR analysis in this study indicates that the encapsulation process involves not only physical entrapment but also secondary interactions (e.g., hydrogen bonding) between the extract and the wall materials, which corroborates the high encapsulation efficiency and the structural stability demonstrated in Section 2.4.2 and Section 2.5.1.

#### 2.5.3. Thermal Stability

In all previous analyses (encapsulation yield, efficiency, moisture, water activity, hygroscopicity, particle size, antioxidant and antibacterial activities), the results refer exclusively to microcapsules prepared with maltodextrin (MD) combined with a small proportion of sodium caseinate (SC). In the present section, thermogravimetric analysis (TGA/DTG) was extended to each individual wall component (MD and SC), their physical mixture (MD–SC), and the final spray-dried extract (SDE), in order to better assess the specific contribution of each material to the overall thermal stability of the encapsulated system.

Thermogravimetric analysis (TGA) was carried out to assess the thermal stability and decomposition behavior of the studied samples, namely the unencapsulated extract (E), maltodextrin (MD), sodium caseinate (SC), the MD–SC mixture, and the spray-dried microcapsules containing the extract (SDE). The analyses were performed under a heating program ranging from 20 to 600 °C, allowing the identification of mass loss events and thermal degradation stages. Complementary derivative thermogravimetric (DTG) curves were used to determine the maximum degradation temperatures associated with each mass loss step.

**Figure 5:**
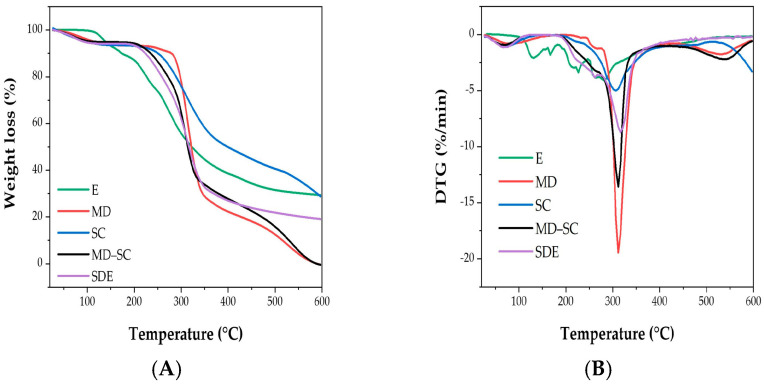
Thermogravimetric (TG, (**A**)) and derivative thermogravimetric (DTG, (**B**)) curves of the studied samples: unencapsulated ethanolic extract of *A. herba-alba* (E), maltodextrin powder (MD), sodium caseinate powder (SC), maltodextrin–sodium caseinate mixture (MD–SC), and spray-dried encapsulated extract (SDE).

According to the TGA and DTG curves (Figure 5A,B), all samples exhibited three stages of thermal degradation, corresponding to successive phases of mass loss.

The first mass loss occurred at ~100 °C for the unencapsulated extract (E), corresponding to solvent evaporation and the volatilization of residual compounds, and accounted for 8.44% of the initial mass [103]. For MD, SC, MD–SC, and SDE, this first stage appeared at slightly lower temperatures (~80 °C), associated with the evaporation of free and bound water involved in hydrogen bonding [119]. Similar results were reported by Yu et al. [66], who observed endothermic peaks related to structural water release between 80 and 150 °C.

The second stage was observed at 228.40 °C for the unencapsulated extract (E), corresponding to a 17.61% mass loss due to phenolic compound degradation. This result is in agreement with Cassol and Noreña [103], who attributed a similar loss at ~229 °C to phenolic decomposition. In contrast, MD, SC, and MD–SC exhibited major weight losses of 71.07%, 53.28%, and 67.09%, respectively, at ~307–313 °C, related to polysaccharide decomposition [106] and protein denaturation [128]. Importantly, the encapsulated extract (SDE) underwent its second degradation step at a higher temperature (261.70 °C) than the unencapsulated extract, with a 20.17% weight loss, indicating enhanced thermal stability. Comparable results were reported by Nunes et al. [129], who demonstrated that spray-dried *Ilex paraguariensis* leaf extracts encapsulated with maltodextrin exhibited greater thermogravimetric stability than the uncoated extracts.

The third stage for the unencapsulated extract (E) occurred at 264.00 °C, with a major weight loss of 44.65%, confirming its limited stability. By contrast, MD, SC, and MD–SC degraded later, at 531.70 °C, 590.00 °C, and 540.10 °C, with weight losses of 22.71%, 13.65%, and 28.36%, respectively. These results are consistent with Castro-López et al. [130], who reported that the final degradation of wall materials such as tragacanth gum (TG) and carboxymethylcellulose (CMC) occurred above 500 °C, corresponding to the breakdown of strongly bonded functional groups. For SDE, the third stage occurred at 316.90 °C, with a mass loss of 54.94%, again confirming its improved thermal resistance compared to the unencapsulated extract.

Overall, these findings demonstrate that the encapsulation of *A. herba-alba* ethanolic extract increases its thermal stability, delaying decomposition to higher temperatures and thereby enhancing its potential for incorporation into food and pharmaceutical products.

## 3. Materials and Methods

### 3.1. Chemicals

Sodium carbonate, Folin–Ciocalteu phenol reagent, copper chloride, iron chloride, potassium persulfate, potassium ferricyanide, ammonium acetate, sodium phosphate dibasic, sodium phosphate monobasic, trichloroacetic acid (TCA), gallic acid, o-phenanthroline, neocuproine, ABTS (2,2′-azino-bis(3-ethylbenzothiazoline-6-sulfonic acid)), DPPH (2,2-diphenyl-1-picrylhydrazyl), butylated hydroxytoluene (BHT), butylated hydroxyanisole (BHA), and dimethyl sulfoxide were purchased from Sigma-Aldrich (Steinheim, Germany). Ethanol (99%) was obtained from VWR Chemicals BDH (Briare, France). Maltodextrin (DE 19) was provided by Roquette-Frères SA (Lestrem, France), and sodium caseinate (92% protein) by Acros Organics (Geel, Belgium). Tryptone Soy Broth (TSB) and Tryptone Soy Agar (TSA) were purchased from Biokar Diagnostics (Beauvais, France).

### 3.2. Plant Material

The aerial parts of *Artemisia herba-alba* Asso. were collected in March 2023 from the Biskra region (Algeria). A reference specimen was deposited under voucher number AP2000 in the herbarium of the Scientific and Technical Research Centre for Arid Regions (CRSTRA, Biskra, Algeria).

Freshly harvested samples were transported to the laboratory, air-dried in the dark for 15 days, and then ground to a fine powder using a coffee grinder. The powder was stored in amber glass bottles at −18 °C until further use.

### 3.3. Extraction of Phenolic Compounds

Phenolic compounds were extracted from *A. herba-alba* using an ultrasound-assisted method. Plant material was mixed with solvent (ethanol/water, 80/20 *v*/*v*) at a ratio of 0.5:10 (g/mL) and sonicated for 10 min at 45 °C in an ultrasonic bath (Jeken TUC-100, Dongguan, China) operating at 40 kHz and 240 W. The mixture was centrifuged (5000 rpm, 10 min), and the supernatant was filtered through Whatman No. 1 paper from Sigma-Aldrich (Steinheim, Germany). The extract was concentrated using a rotary evaporator (Rotavapor R-100, Büchi, Flawil, Switzerland) at 40 °C, and then freeze-dried with a laboratory-scale freeze-dryer (Lyovapor™ L-200, Büchi, Rungis, France). The resulting freeze-dried extract was stored at −18 °C until further analysis.

### 3.4. Encapsulation of Phenolic Compounds

Spray-drying was performed using maltodextrin (DE 19) and sodium caseinate as encapsulating agents. First, a 0.5% (*w*/*v*) solution of *A. herba-alba* extract was prepared in distilled water under continuous stirring (30 min, room temperature, dark conditions). Maltodextrin and sodium caseinate were then gradually added to reach final concentrations of 19% and 0.5% (*w*/*v*), respectively, to prevent aggregation. The mixture was homogenized with an Ultra-Turrax (IKA T-18 basic, Staufen, Germany) at 10,000 rpm for 5 min.

The feed solution was spray-dried using a Büchi Mini Spray-Dryer B-290 (Flawil, Switzerland) equipped with a 0.5 mm nozzle, operating at a flow rate of 8.33 mL/min. Inlet and outlet air temperatures were set at 150 ± 2 °C and 80 ± 5 °C, respectively. The microcapsule powder collected at the cyclone outlet was stored in airtight containers at −18 °C in the dark until analysis.

### 3.5. Determination of Phenolic Content

#### 3.5.1. Total Phenolic Content (TPC)

The TPC of unencapsulated extract (E) and spray-dried encapsulated extract (SDE) was determined using gallic acid as a standard. For encapsulated samples, a pre-treatment step was performed to disrupt the microcapsules. Briefly, 40 mg of SDE was suspended in 200 µL of distilled water in a 2 mL Eppendorf tube, vortexed for 7 min, and then mixed with 800 µL of absolute ethanol. The suspension was stirred and centrifuged at 1000× *g* for 5 min at 25 ± 2 °C. The supernatant was used for TPC determination and subsequent analyses.

The Folin–Ciocalteu method was performed according to Zahnit et al. [131]. Briefly, 20 µL of each sample was mixed with 100 µL of diluted Folin–Ciocalteu reagent (1:10 with distilled water). After 4 min, 75 µL of sodium carbonate solution (7.5%, *w*/*v*) was added. Following 2 h of incubation at room temperature in the dark, absorbance was measured at 765 nm using a 96-well microplate reader (Thermo Fisher Scientific, A51119600C, Illkirch-Graffenstaden, France).

Results were expressed as µg gallic acid equivalents per mg dry extract (µg GAE/mg DE), based on a gallic acid calibration curve (12.5–150 µg/mL, y = 0.0067x + 0.0942, R^2^ = 0.9981).

#### 3.5.2. Surface Phenolic Content (SPC)

SPC of SDE was determined following Zahnit et al. [131]. A total of 40 mg of microcapsules was suspended in 1 mL of absolute ethanol, vortexed for 10 s, and centrifuged at 10,000× *g* for 1 min at room temperature. The supernatant was used for SPC determination.

SPC values were expressed as µg GAE/mg DE, calculated from a gallic acid calibration curve (12.5–150 µg/mL, y = 0.008x + 0.0687, R^2^ = 0.999).

### 3.6. Evaluation of Antioxidant Activity

The antioxidant activity of unencapsulated ethanolic extract of *A. herba-alba* (E) and the spray-dried microcapsule powder containing the same extract (SDE) was evaluated using five assays: ABTS•^+^ (2,2′-azino-bis(3-ethylbenzothiazoline-6-sulfonic acid)) and DPPH• (2,2-diphenyl-1-picrylhydrazyl) radical scavenging assays, the cupric reducing antioxidant capacity (CUPRAC), the ferric reducing power assay, and the phenanthroline assay.

Serial two-fold dilutions of the unencapsulated extract were prepared in 80% ethanol to obtain concentrations ranging from 200 to 3.125 µg/mL. An identical dilution series was prepared using the supernatant recovered from microcapsules, which contained the released phenolic compounds. Butylated hydroxytoluene (BHT) and butylated hydroxyanisole (BHA) served as antioxidant standards.

#### 3.6.1. ABTS•^+^ Radical Scavenging Assay

The ABTS•^+^ radical scavenging activity was determined according to Khenifi et al. [132], in triplicate. The inhibition percentage was calculated using Equation (1):

(1) $$Inhibition\;\;(\%)=\frac{Ac-As}{Ac}\times 100$$

where *Ac* is the absorbance of the control (reaction mixture containing ABTS•^+^ solution and solvent) and *As* is the absorbance of the sample mixture.

Results were expressed as IC_50_ (µg/mL), defined as the concentration required to inhibit 50% of ABTS•^+^ radicals, determined graphically from inhibition curves.

#### 3.6.2. DPPH• Radical Scavenging Assay

The DPPH• scavenging activity was assessed following the method of Khenifi et al. [132], in triplicate. The inhibition percentage was calculated using Equation (2):

(2) $$Inhibition\;\left(\%\right)=\frac{A_{0}-As}{A_{0}}\times 100$$

where *A*_0_ is the absorbance of the control (reaction mixture with DPPH• solution and solvent) and *A*_0_ is the absorbance of the sample reaction mixture.

Results were expressed as IC_50_ (µg/mL), corresponding to the concentration required to inhibit 50% of DPPH• radicals.

#### 3.6.3. CUPRAC Assay

The cupric reducing antioxidant capacity (CUPRAC) was determined according to Benouchenne et al. [133], in triplicate. Antioxidant capacity was expressed as A_0.50_ (µg/mL), the concentration at which the absorbance of the reaction medium reached 0.50, estimated from the absorbance–concentration curve.

#### 3.6.4. Reducing Power Assay

The ferric reducing power of the samples was determined according to the method of Djermane et al. [134]. Antioxidant capacity was expressed as A_0.50_ (µg/mL), corresponding to the concentration at which the absorbance of the reaction mixture reached 0.50.

#### 3.6.5. Phenanthroline Assay

The phenanthroline assay was carried out as described by Bendjedid et al. [135]. Results were expressed as A_0.50_ (µg/mL), defined as the concentration of the sample that produced an absorbance intensity of 0.50 in the reaction mixture.

### 3.7. Evaluation of Antibacterial Activity

#### 3.7.1. Source and Selection of Bacterial Strains

The antibacterial activity of unencapsulated (E) and encapsulated (SDE) ethanolic extracts of *A. herba-alba* was evaluated against foodborne bacteria. Five strains were selected, obtained from the German Collection of Microorganisms (DSMZ, Braunschweig, Germany): two Gram-positive strains (*Listeria innocua* DSM 20640 and *Brochothrix thermosphacta* DSM 20171) and three Gram-negative strains (*Pseudomonas aeruginosa* CIP 103467, *Salmonella enterica* DSM 11320, and *Escherichia coli* DSM 613). Strains were stored at −20 °C in Tryptone Soy Broth (TSB) supplemented with 15% (*v/v*) glycerol [136].

#### 3.7.2. Inoculum Preparation

Bacterial strains were revived and cultured in TSB prior to testing, in accordance with the method proposed by Guebebia et al. [136]. One milliliter of thawed stock culture was inoculated into 9 mL of TSB and incubated for 8 h at 37 °C for all strains, except *B. thermosphacta*, which was incubated at 25 °C (optimum growth temperature).

Subsequently, 1 mL of each pre-culture was transferred into 9 mL of fresh TSB and incubated for 16 h under the same conditions (37 °C for *L. innocua*, *P. aeruginosa*, *S. enterica*, and *E. coli*; 25 °C for *B. thermosphacta*). A third transfer was performed by inoculating 1 mL of culture into 9 mL of TSB, followed by incubation for 5 h at the respective optimum temperature. The resulting cultures were adjusted to a final concentration of 10^6^ CFU/mL.

#### 3.7.3. Agar Well Diffusion Assay

The antibacterial activity of E and SDE was assessed using the agar well diffusion method, according to the previously used protocol [136]. Briefly, 1 mL of bacterial inoculum was evenly spread onto Tryptone Soy Agar (TSA) plates under aseptic conditions. Excess inoculum was removed, and 6 mm wells were created in the agar using sterile Pasteur pipettes.

For the unencapsulated extract, a stock solution was prepared at 10 mg/mL in DMSO, and 100 µL was dispensed into each well. DMSO served as the negative control, while chloramphenicol (30 µg/well) was used as the positive control. For the encapsulated extract, 40 mg of spray-dried microcapsules (containing an equivalent concentration of extract) were placed directly into the wells.

The plates were pre-incubated at 4 °C for 1 h to allow diffusion, then incubated at the appropriate temperature for each strain for 24 h. All assays were performed in triplicate. Antibacterial activity was expressed as the diameter of inhibition zones (mm) measured with a caliper.

### 3.8. Determination of Physicochemical Characteristics of Powder

#### 3.8.1. Determination of Encapsulation Yield

Encapsulation yield was determined according to Ocak [112], as the percentage ratio between the weight of microcapsules recovered after spray-drying and the weight of solids in the initial feed solution:

(3) $$Encapsulation\;yield\;(\%)=\frac{Wm}{Wis}\times 100$$

where *Wm* is the mass of collected microcapsules (g), and *Wis* is the total mass of solids in the initial feed solution.

#### 3.8.2. Determination of Encapsulation Efficiency

Encapsulation efficiency of phenolic compounds (EE) was determined in triplicate following Pashazadeh et al. [42], using Equation (4):

(4) $$Encapsulation\;efficiency\;(\%)=\frac{TPC-SPC}{TPC}\times 100$$

where *TPC* is the total phenolic content in microcapsules, and *SPC* is the surface phenolic content.

#### 3.8.3. Moisture Content Measurement

Moisture was determined according to Ocak [112], in triplicate. Briefly, 500 mg of SDE was placed in a pre-weighed aluminum dish and dried in an oven (Memmert UNB 400, Büchenbach, Germany) at 105 ± 3 °C for 24 h until constant weight. Samples were cooled for 30 min in a silica gel desiccator before weighing. Moisture content was calculated as follows:

(5) $$Moisture\;\left(\%\right)=\frac{W1-\left(W3-W2\right)}{W1}\times 100$$

where *W*1 is the mass of the sample before drying, *W*2 the mass of the empty dish, and *W*3 the mass of the dish containing the dried sample.

#### 3.8.4. Water Activity Measurement

Water activity (a_w_) of SDE was measured at 25 °C using a water activity meter (Novasina LabSwift-aw, Lachen, Switzerland), in triplicate.

#### 3.8.5. Hygroscopicity Measurement

Hygroscopicity was determined according to Bhagya Raj and Dash [52], with slight modifications. In brief, 500 mg of SDE was placed in a pre-weighed aluminum dish and conditioned in a silica gel desiccator for 48 h. Samples were weighed, then transferred to a desiccator containing saturated NaCl solution (75 ± 2% RH) and stored for 7 days. Hygroscopicity was calculated as follows:

(6) $$Hygroscopicity\;\left(\%\right)=\frac{W2-W1}{W1}\times 100$$

where *W*1 is the mass of the conditioned sample, and *W*2 is the mass after storage at 75 ± 2% RH.

#### 3.8.6. Particle Size Measurement

Particle size distribution of SDE was analyzed using a laser diffraction instrument (Mastersizer 3000, Malvern Instruments, Malvern, UK). Microcapsules were dispersed in ethanol (refractive index = 1.361) under continuous stirring to avoid aggregation. Measurements were performed in triplicate, and results were expressed as the volume-weighted mean diameter D [4,3] [136].

### 3.9. Determination of Structural Characterstics of Powder

#### 3.9.1. Scanning Electron Microscopy (SEM)

The surface morphology of spray-dried microcapsules containing *A. herba-alba* extract (SDE) was examined by scanning electron microscopy (SEM) (FEI Quanta 250 FEG, Eindhoven, The Netherlands). Images were acquired at an accelerating voltage of 10 kV with magnifications ranging from ×400 to ×3000.

#### 3.9.2. Attenuated Total Reflectance–Fourier Transform Infrared (ATR-FTIR) Spectroscopy

ATR-FTIR spectroscopy was performed on the following samples: ethanolic extract (E), maltodextrin (MD) powder, sodium caseinate (SC) powder, MD–SC mixture powder, and spray-dried microcapsules containing *A. herba-alba* extract (SDE). Measurements were carried out using a Nicolet iS50 FTIR spectrophotometer (Thermo Fisher Scientific, Waltham, MA, USA) equipped with an ATR accessory. Spectra were recorded in the 4000–400 cm^−1^ range at a resolution of 4 cm^−1^, averaging 16 scans per sample. Data were processed and plotted using OriginPro^®^ 18 software.

#### 3.9.3. Thermogravimetric and Derivative Thermogravimetric Analysis (TGA/DTG)

Thermogravimetric analysis (TGA) was conducted using a Netzsch TG 209 analyzer (Selb, Germany). Approximately 10 mg of each sample (E, MD powder, SC powder, MD–SC complex powder, and SDE) was placed in an alumina crucible and heated from 20 to 600 °C at a rate of 10 °C/min under a nitrogen atmosphere (20 mL/min). Weight loss profiles were obtained using NETZSCH Proteus 6.1.0 software, and derivative thermogravimetric (DTG) curves were generated to determine the temperatures corresponding to maximum weight loss rates.

### 3.10. Statistical Analysis

All experiments were performed in triplicate, and results were expressed as mean ± standard deviation (SD). Data were subjected to one-way analysis of variance (ANOVA) followed by Tukey’s post hoc test at a 95% confidence level, using Minitab^®^ version 18 (Minitab Inc., State College, PA, USA).

## 4. Conclusions

In this study, the ethanolic extract of the aerial parts of *A. herba-alba* was successfully encapsulated by spray-drying using maltodextrin (MD) in combination with sodium caseinate (SC) as wall materials, with the aim of improving the stability and functionality of its phenolic compounds. The optimized MD–SC formulation achieved a high encapsulation yield (69.40%) and an encapsulation efficiency exceeding 96%, confirming the suitability of this carrier system.

The resulting MD–SC microcapsule powder exhibited favorable physicochemical properties, including low moisture content (4.34%), moderate water activity (0.415), and acceptable hygroscopicity (12.67%), ensuring both biochemical and microbiological stability during storage. The microcapsules had a fine particle size (10.05 ± 0.08 µm), compatible with food applications without impairing texture or sensory attributes.

In terms of functionality, encapsulation preserved the antioxidant potential of the extract, although activity was reduced in some assays compared to the free extract, likely due to partial phenolic degradation and reduced accessibility. Nevertheless, the encapsulated extract retained significant bioactivity. The MD–SC microcapsules also showed antibacterial activity, particularly against *Brochothrix thermosphacta* and *Pseudomonas aeruginosa*, with inhibition zones comparable to values reported in the literature for other plant extracts.

Structural analyses supported these findings. SEM imaging revealed spherical, non-agglomerated microcapsules with mostly smooth surfaces, without cracks, consistent with high encapsulation efficiency. ATR-FTIR spectroscopy confirmed the incorporation of phenolic compounds into the MD–SC matrix through hydrogen bonding and other non-covalent interactions. TGA/DTG analysis demonstrated that encapsulation enhanced the thermal stability of the extract, with degradation occurring at higher temperatures compared to the unencapsulated form, further supporting its suitability for processing and storage.

Overall, these results indicate that spray-drying microencapsulation with the MD–SC carrier system is a promising strategy to preserve, stabilize, and deliver phenolic compounds from *A. herba-alba*. The stable and functional microcapsule powder obtained in this study could serve as a natural preservative with potential applications in food, nutraceutical, and pharmaceutical industries, thereby contributing to the development of clean-label, bioactive-enriched products.

## Figures and Tables

**Table 1:** Inhibition zone diameters (mm) of the antibacterial activity of the unencapsulated ethanolic extract of *A. herba-alba* (E), the spray-dried encapsulated extract (SDE), and the reference antibiotic chloramphenicol against *Listeria innocua*, *Brochothrix thermosphacta*, *Pseudomonas aeruginosa*, *Salmonella enterica*, and *Escherichia coli*.

Bacteria	Inhibition Zone Diameter for (E) Sample (mm)	Inhibition Zone Diameter for (SDE) Sample (mm)	Inhibition Zone Diameter for Chloramphenicol (mm)
*Listeria innocua*	-	-	29.21 ± 0.16
*Brochothrix thermosphacta*	11.56 ± 0.49	8.92 ± 0.05	32.93 ± 0.59
*Pseudomonas aeruginosa*	11.00 ± 0.72	10.59 ± 0.12	12.00 ± 0.59
*Salmonella enterica*	-	-	30.12 ± 0.03
*Escherichia coli*	-	-	27.78 ± 0.47

The data of measured zones are presented as mean ± standard deviation (*n* = 3); (-): no inhibition zone determined.

**Table 2:** Physicochemical properties of spray-dried microcapsules of *A. herba-alba* extract (SDE).

Physicochemical Properties	SDE
Encapsulation yield (%)	69.40 ± 0.25
Encapsulation efficiency (%)	96.39 ± 0.11
Moisture (%)	4.34 ± 0.10
Water activity	0.415 ± 0.01
Hygroscopicity (%)	12.67 ± 0.75
Particle size D [4,3] (µm)	10.05 ± 0.08

Results are expressed as mean ± standard deviation (*n* = 3).

## Data Availability

Data are contained within the article.

## References

[1] Dhifallah A., Selmi H., Ouerghui A., Sammeri H., Aouini D., Rouissi H. (2022). Comparative Study of Phenolic Compounds and Antiradical Activities of Four Extracts of Tunisian Artemisia Herba Alba. Pharm. Chem. J..

[2] Khlifi D., Sghaier R.M., Amouri S., Laouini D., Hamdi M., Bouajila J. (2013). Composition and Anti-Oxidant, Anti-Cancer and Anti-Inflammatory Activities of *Artemisia herba-alba*, *Ruta chalpensis* L. and *Peganum harmala* L.. Food Chem. Toxicol..

[3] Mrabti H.N., Hachlafi N.E., Al-Mijalli S.H., Jeddi M., Elbouzidi A., Abdallah E.M., Flouchi R., Assaggaf H., Qasem A., Zengin G. (2023). Phytochemical Profile, Assessment of Antimicrobial and Antioxidant Properties of Essential Oils of *Artemisia herba-alba* Asso., and *Artemisia dracunculus* L.: Experimental and Computational Approaches. J. Mol. Struct..

[4] Bertella A., Benlahcen K., Abouamama S., Pinto D.C.G.A., Maamar K., Kihal M., Silva A.M.S. (2018). *Artemisia herba-alba* Asso. Essential Oil Antibacterial Activity and Acute Toxicity. Ind. Crops Prod..

[5] Mighri H., Hajlaoui H., Akrout A., Najjaa H., Neffati M. (2009). Antimicrobial and Antioxidant Activities of *Artemisia herba-alba* Essential Oil Cultivated in Tunisian Arid Zone. Comptes Rendus Chim..

[6] Mohammed J.K., Mahdi A.A., Ma C., Elkhedir A.E., Al-Maqtari Q.A., Al-Ansi W., Mahmud A., Wang H. (2021). Application of Argun Fruit Polysaccharide in Microencapsulation of Citrus Aurantium L. Essential Oil: Preparation, Characterization, and Evaluating the Storage Stability and Antioxidant Activity. J. Food Meas. Charact..

[7] Younsi F., Trimech R., Boulila A., Ezzine O., Dhahri S., Boussaid M., Messaoud C. (2016). Essential Oil and Phenolic Compounds of *Artemisia herba-alba* (Asso.): Composition, Antioxidant, Antiacetylcholinesterase, and Antibacterial Activities. Int. J. Food Prop..

[8] Adel K., Zied Z., Ahmed B.K., Neacute Ji G., Mohamed D., Radhouane G. (2011). Chemical Constituents and Antioxidant Activity of the Essential Oil from Aerial Parts of *Artemisia herba-alba* Grown in Tunisian Semi-Arid Region. Afr. J. Biotechnol..

[9] Souhila T., Fatma Zohra B., Tahar H.S. (2019). Identification and Quantification of Phenolic Compounds of *Artemisia herba-alba* at Three Harvest Time by HPLC–ESI–Q-TOF–MS. Int. J. Food Prop..

[10] Sendi N., Mkadmini-Hammi K., Ben Mansour R., Selmi S., Trabelsi N., Isoda H., Ksouri R., Megdiche-Ksouri W. (2020). Simultaneous Optimization of Ultrasound-Assisted Extraction of Flavonoid Compounds and Antiradical Activity from *Artemisia herba-alba* Using Response Surface Methodology. Prep. Biochem. Biotechnol..

[11] Bourgou S., Bettaieb Rebey I., Mkadmini K., Isoda H., Ksouri R., Ksouri W.M. (2017). LC-ESI-TOF-MS and GC-MS Profiling of *Artemisia herba-alba* and Evaluation of Its Bioactive Properties. Food Res. Int..

[12] Elwardani H., Oubihi A., Haida S., Ez-Zriouli R., Kabous K.E., Ouhssine M. (2024). Seasonal Variation in Essential Oil Composition of *Artemisia herba-alba* and Their Effects on Antioxidant, Antibacterial, and Antifungal Activities. Chem. Data Collect..

[13] Khojasteh A., Mirjalili M.H., Alcalde M.A., Cusido R.M., Eibl R., Palazon J. (2020). Powerful Plant Antioxidants: A New Biosustainable Approach to the Production of Rosmarinic Acid. Antioxidants.

[14] Tajner-Czopek A., Gertchen M., Rytel E., Kita A., Kucharska A.Z., Sokół-Łętowska A. (2020). Study of Antioxidant Activity of Some Medicinal Plants Having High Content of Caffeic Acid Derivatives. Antioxidants.

[15] Chamali S., Bendaoud H., Bouajila J., Camy S., Saadaoui E., Condoret J.-S., Romdhane M. (2023). Optimization of Accelerated Solvent Extraction of Bioactive Compounds from Eucalyptus Intertexta Using Response Surface Methodology and Evaluation of Its Phenolic Composition and Biological Activities. J. Appl. Res. Med. Aromat. Plants.

[16] Alves D., Pinho E., Ho T.M., Yoshii H., Terao K., Bhandari B.R. (2021). Encapsulation of Polyphenols, Plant Bioactive Compounds. Functionality of Cyclodextrins in Encapsulation for Food Applications.

[17] Garavand F., Jalai-Jivan M., Assadpour E., Jafari S.M. (2021). Encapsulation of Phenolic Compounds within Nano/Microemulsion Systems: A Review. Food Chem..

[18] Sansone F., Mencherini T., Picerno P., d’Amore M., Aquino R.P., Lauro M.R. (2011). Maltodextrin/Pectin Microparticles by Spray Drying as Carrier for Nutraceutical Extracts. J. Food Eng..

[19] Stanković N., Mihajilov-Krstev T., Zlatković B., Stankov-Jovanović V., Mitić V., Jović J., Čomić L., Kocić B., Bernstein N. (2016). Antibacterial and Antioxidant Activity of Traditional Medicinal Plants from the Balkan Peninsula. NJAS—Wagening. J. Life Sci..

[20] Mighri H., Akrout A., Bennour N., Eljeni H., Zammouri T., Neffati M. (2019). LC/MS Method Development for the Determination of the Phenolic Compounds of Tunisian Ephedra Alata Hydro-Methanolic Extract and Its Fractions and Evaluation of Their Antioxidant Activities. S. Afr. J. Bot..

[21] Hcini K., Lozano-Pérez A.A., Luis Cenis J., Quílez M., José Jordán M. (2021). Extraction and Encapsulation of Phenolic Compounds of Tunisian Rosemary (*Rosmarinus officinalis* L.) Extracts in Silk Fibroin Nanoparticles. Plants.

[22] Alvarez Gaona I.J., Fanzone M.L., Galmarini M.V., Chirife J., Ferreras-Charro R., García-Estévez I., Escribano-Bailón M.T. (2022). Encapsulation of Phenolic Compounds by Spray Drying of Ancellotta and Aspirant Bouchet Wines to Produce Powders with Potential Use as Natural Food Colorants. Food Biosci..

[23] da Rosa C.G., Borges C.D., Zambiazi R.C., Rutz J.K., da Luz S.R., Krumreich F.D., Benvenutti E.V., Nunes M.R. (2014). Encapsulation of the Phenolic Compounds of the Blackberry (*Rubus Fruticosus*). LWT—Food Sci. Technol..

[24] Mehta N., Kumar P., Verma A.K., Umaraw P., Kumar Y., Malav O.P., Sazili A.Q., Domínguez R., Lorenzo J.M. (2022). Microencapsulation as a Noble Technique for the Application of Bioactive Compounds in the Food Industry: A Comprehensive Review. Appl. Sci..

[25] Ferro D.M., Müller C.M.O., Ferreira S.R.S. (2020). Photostability and Characterization of Spray-Dried Maltodextrin Powders Loaded with Sida Rhombifolia Extract. Biocatal. Agric. Biotechnol..

[26] Vergara L.P., Dos Santos Hackbart H.C., Jansen Alves C., Reissig G.N., Wachholz B.S., Borges C.D., Chim J.F., Zambiazi R.C. (2023). Encapsulation of Phenolic Compounds through the Complex Coacervation Technique for the Enrichment of Diet Chewable Candies. Food Biosci..

[27] Klein T., Longhini R., Bruschi M.L., De Mello J.C.P. (2015). Microparticles Containing Guaraná Extract Obtained by Spray-Drying Technique: Development and Characterization. Rev. Bras. Farmacogn..

[28] Labuschagne P. (2018). Impact of Wall Material Physicochemical Characteristics on the Stability of Encapsulated Phytochemicals: A Review. Food Res. Int..

[29] Saenz C., Tapia S., Chavez J., Robert P. (2009). Microencapsulation by Spray Drying of Bioactive Compounds from Cactus Pear (*Opuntia ficus-indica*). Food Chem..

[30] Pudziuvelyte L., Marksa M., Jakstas V., Ivanauskas L., Kopustinskiene D.M., Bernatoniene J. (2019). Microencapsulation of Elsholtzia Ciliata Herb Ethanolic Extract by Spray-Drying: Impact of Resistant-Maltodextrin Complemented with Sodium Caseinate, Skim Milk, and Beta-Cyclodextrin on the Quality of Spray-Dried Powders. Molecules.

[31] Mainente F., Piovan A., Zanoni F., Chignola R., Cerantola S., Faggin S., Giron M.C., Filippini R., Seraglia R., Zoccatelli G. (2022). Spray-Drying Microencapsulation of an Extract from Tilia Tomentosa Moench Flowers: Physicochemical Characterization and in Vitro Intestinal Activity. Plant Foods Hum. Nutr..

[32] Savan E.K., Ramadan M.F. (2023). Encapsulation of Cardamom Extracts. Cardamom (*Elettaria cardamomum*): Production, Processing and Properties.

[33] Ștefănescu B.E., Nemes S.A., Teleky B.E., Călinoiu L.F., Mitrea L., Martău G.A., Szabo K., Mihai M., Vodnar D.C., Crișan G. (2022). Microencapsulation and Bioaccessibility of Phenolic Compounds of Vaccinium Leaf Extracts. Antioxidants.

[34] Eghbal N., Liao W., Dumas E., Azabou S., Dantigny P., Gharsallaoui A. (2022). Microencapsulation of Natural Food Antimicrobials: Methods and Applications. Appl. Sci..

[35] Kuhn F., Santos Dorneles M., Pelayo Zapata Noreña C. (2022). Accelerated Stability Testing and Simulated Gastrointestinal Release of Encapsulated Betacyanins and Phenolic Compounds from Bougainvillea Glabra Bracts Extract. Food Chem..

[36] Moro K.I.B., Bender A.B.B., Da Silva L.P., Penna N.G. (2021). Green Extraction Methods and Microencapsulation Technologies of Phenolic Compounds From Grape Pomace: A Review. Food Bioprocess Technol..

[37] Cilek B., Luca A., Hasirci V., Sahin S., Sumnu G. (2012). Microencapsulation of Phenolic Compounds Extracted from Sour Cherry Pomace: Effect of Formulation, Ultrasonication Time and Core to Coating Ratio. Eur. Food Res. Technol..

[38] Sylla N., Bouyahya A., Taha D., Dakka N., Elhajji H. (2021). Study of the Antioxidant and Antidiabetic Activity in Vitro of Free and Encapsulated Phenolic Compounds of Olive Pomace. Biocatal. Agric. Biotechnol..

[39] Aguiar J., Estevinho B.N., Santos L. (2016). Microencapsulation of Natural Antioxidants for Food Application—The Specific Case of Coffee Antioxidants—A Review. Trends Food Sci. Technol..

[40] Gaber Ahmed G.H., Fernández-González A., Díaz García M.E. (2020). Nano-Encapsulation of Grape and Apple Pomace Phenolic Extract in Chitosan and Soy Protein via Nanoemulsification. Food Hydrocoll..

[41] Herman-Lara E., Rivera-Abascal I., Gallegos-Marín I., Martínez-Sánchez C.E. (2024). Encapsulation of Hydroalcoholic Extracts of Moringa Oleifera Seed through Ionic Gelation. LWT—Food Sci. Technol..

[42] Pashazadeh H., Zannou O., Ghellam M., Koca I., Galanakis C.M., Aldawoud T.M.S. (2021). Optimization and Encapsulation of Phenolic Compounds Extracted from Maize Waste by Freeze-Drying, Spray-Drying, and Microwave-Drying Using Maltodextrin. Foods.

[43] Luca A., Cilek B., Hasirci V., Sahin S., Sumnu G. (2014). Storage and Baking Stability of Encapsulated Sour Cherry Phenolic Compounds Prepared from Micro- and Nano-Suspensions. Food Bioprocess Technol..

[44] Avilés-Betanzos K.A., Cauich-Rodríguez J.V., Ramírez-Sucre M.O., Rodríguez-Buenfil I.M. (2023). Optimization of Spray-Drying Conditions of Microencapsulated Habanero Pepper (*Capsicum chinense* Jacq.) Extracts and Physicochemical Characterization of the Microcapsules. Processes.

[45] Silva P.I., Stringheta P.C., Teófilo R.F., de Oliveira I.R.N. (2013). Parameter Optimization for Spray-Drying Microencapsulation of Jaboticaba (*Myrciaria jaboticaba*) Peel Extracts Using Simultaneous Analysis of Responses. J. Food Eng..

[46] Simon-Brown K., Solval K.M., Chotiko A., Alfaro L., Reyes V., Liu C., Dzandu B., Kyereh E., Goldson Barnaby A., Thompson I. (2016). Microencapsulation of Ginger (*Zingiber officinale*) Extract by Spray Drying Technology. LWT—Food Sci. Technol..

[47] Tran T.T., Nguyen V.H. (2018). Effects of Spray-Drying Temperatures and Carriers on Physical and Antioxidant Properties of Lemongrass Leaf Extract Powder. Beverages.

[48] Gomes W.F., França F.R.M., Denadai M., Andrade J.K.S., Da Silva Oliveira E.M., De Brito E.S., Rodrigues S., Narain N. (2018). Effect of Freeze- and Spray-Drying on Physico-Chemical Characteristics, Phenolic Compounds and Antioxidant Activity of Papaya Pulp. J. Food Sci. Technol..

[49] Muñoz-Shugulí C., Vidal C.P., Cantero-López P., Lopez-Polo J. (2021). Encapsulation of Plant Extract Compounds Using Cyclodextrin Inclusion Complexes, Liposomes, Electrospinning and Their Combinations for Food Purposes. Trends Food Sci. Technol..

[50] Shi L., Zhou J., Guo J., Gladden I., Kong L. (2021). Starch Inclusion Complex for the Encapsulation and Controlled Release of Bioactive Guest Compounds. Carbohydr. Polym..

[51] Ayar-Sumer E.N., Nyambe C., Hashim M.A., Altin-Yavuzarslan G., El-Messery T.M., Ozçelik B. (2024). Optimizing Encapsulation of Black Carrot Extract Using Complex Coacervation Technique: Maximizing the Bioaccessibility and Release Kinetics in Different Food Matrixes. LWT—Food Sci. Technol..

[52] Bhagya Raj G.V.S., Dash K.K. (2022). Microencapsulation of Betacyanin from Dragon Fruit Peel by Complex Coacervation: Physicochemical Characteristics, Thermal Stability, and Release Profile of Microcapsules. Food Biosci..

[53] Figueroa-Robles A., Antunes-Ricardo M., Guajardo-Flores D. (2021). Encapsulation of Phenolic Compounds with Liposomal Improvement in the Cosmetic Industry. Int. J. Pharm..

[54] Jahanfar S., Gahavami M., Khosravi-Darani K., Jahadi M., Mozafari M.R. (2021). Entrapment of Rosemary Extract by Liposomes Formulated by Mozafari Method: Physicochemical Characterization and Optimization. Heliyon.

[55] Parhizkary M., Jafari S.M., Assadpour E., Enayati A., Kashiri M. (2024). Pea Protein-Coated Nanoliposomal Encapsulation of Jujube Phenolic Extract with Different Stabilizers; Characterization and In Vitro Release. Food Chem. X.

[56] Chanioti S., Katsouli M., Tzia C. (2021). Novel Processes for the Extraction of Phenolic Compounds from Olive Pomace and Their Protection by Encapsulation. Molecules.

[57] Rosa A.D., Secco M.C., De Cezaro A.M., Fischer B., Cansian R.L., Junges A., Franceschi E., Backes G.T., Valduga E. (2023). Encapsulation of Olive Leaf (*Olea europaea*) Extract Using Solution-Enhanced Dispersion by Supercritical Fluids (SEDS) Technique. J. Supercrit. Fluids.

[58] Wen P., Zong M.H., Linhardt R.J., Feng K., Wu H. (2017). Electrospinning: A Novel Nano-Encapsulation Approach for Bioactive Compounds. Trends Food Sci. Technol..

[59] Bratovcic A., Suljagic J. (2019). Micro- and Nano-Encapsulation in Food Industry. Croat. J. Food Sci. Technol..

[60] Rajapaksha D.S.W., Shimizu N. (2020). Valorization of Spent Black Tea by Recovery of Antioxidant Polyphenolic Compounds: Subcritical Solvent Extraction and Microencapsulation. Food Sci. Nutr..

[61] Aliakbarian B., Sampaio F.C., de Faria J.T., Pitangui C.G., Lovaglio F., Casazza A.A., Converti A., Perego P. (2018). Optimization of Spray Drying Microencapsulation of Olive Pomace Polyphenols Using Response Surface Methodology and Artificial Neural Network. LWT—Food Sci. Technol..

[62] Geranpour M., Assadpour E., Jafari S.M. (2020). Recent Advances in the Spray Drying Encapsulation of Essential Fatty Acids and Functional Oils. Trends Food Sci. Technol..

[63] Di Battista C.A., Constenla D., Ramírez Rigo M.V., Piña J. (2017). Process Analysis and Global Optimization for the Microencapsulation of Phytosterols by Spray Drying. Powder Technol..

[64] Hoyos Merlano N.T., Borroni V., Giménez R.B., Herrera M.L., Gomez-Zavaglia A. (2021). Encapsulation of Hydrophobic Compounds in Sugar Matrixes by Freeze-Drying. Basic Protocols in Encapsulation of Food Ingredients.

[65] Leyva-Jiménez F.J., Lozano-Sánchez J., Cádiz-Gurrea M.D.L.L., Fernández-Ochoa Á., Arráez-Román D., Segura-Carretero A. (2020). Spray-Drying Microencapsulation of Bioactive Compounds from Lemon Verbena Green Extract. Foods.

[66] Yu F., Li Z., Zhang T., Wei Y., Xue Y., Xue C. (2017). Influence of Encapsulation Techniques on the Structure, Physical Properties, and Thermal Stability of Fish Oil Microcapsules by Spray Drying. J. Food Process Eng..

[67] Paini M., Aliakbarian B., Casazza A.A., Lagazzo A., Botter R., Perego P. (2015). Microencapsulation of Phenolic Compounds from Olive Pomace Using Spray Drying: A Study of Operative Parameters. LWT—Food Sci. Technol..

[68] Sadiq U., Gill H., Chandrapala J., Shahid F. (2022). Influence of Spray Drying on Encapsulation Efficiencies and Structure of Casein Micelles Loaded with Anthraquinones Extracted from Aloe Vera Plant. Appl. Sci..

[69] Zhu J., Li X., Liu L., Li Y., Qi B., Jiang L. (2022). Preparation of Spray-Dried Soybean Oil Body Microcapsules Using Maltodextrin: Effects of Dextrose Equivalence. LWT—Food Sci. Technol..

[70] Desai N.M., Haware D.J., Basavaraj K., Murthy P.S. (2019). Microencapsulation of Antioxidant Phenolic Compounds from Green Coffee. Prep. Biochem. Biotechnol..

[71] Bednarska M.A., Janiszewska-Turak E. (2020). The Influence of Spray Drying Parameters and Carrier Material on the Physico-Chemical Properties and Quality of Chokeberry Juice Powder. J. Food Sci. Technol..

[72] Lu W., Yang X., Shen J., Li Z., Tan S., Liu W., Cheng Z. (2021). Choosing the Appropriate Wall Materials for Spray-Drying Microencapsulation of Natural Bioactive Ingredients: Taking Phenolic Compounds as Examples. Powder Technol..

[73] Šturm L., Osojnik Črnivec I.G., Istenič K., Ota A., Megušar P., Slukan A., Humar M., Levic S., Nedović V., Kopinč R. (2019). Encapsulation of Non-Dewaxed Propolis by Freeze-Drying and Spray-Drying Using Gum Arabic, Maltodextrin and Inulin as Coating Materials. Food Bioprod. Process..

[74] Tao Y., Wang P., Wang J., Wu Y., Han Y., Zhou J. (2017). Combining Various Wall Materials for Encapsulation of Blueberry Anthocyanin Extracts: Optimization by Artificial Neural Network and Genetic Algorithm and a Comprehensive Analysis of Anthocyanin Powder Properties. Powder Technol..

[75] Hamed I., Moradi M., Ezati P., O’Higgins L., Meléndez-Martínez A.J., Frleta Matas R., Šimat V., McClements D.J., Jakobsen A.N., Lerfall J. (2023). Encapsulation of Microalgal-Based Carotenoids: Recent Advances in Stability and Food Applications. Trends Food Sci. Technol..

[76] Sekiou O., Boumendjel M., Taibi F., Tichati L., Boumendjel A., Messarah M. (2021). Nephroprotective Effect of Artemisia Herba Alba Aqueous Extract in Alloxan-Induced Diabetic Rats. J. Tradit. Complement. Med..

[77] Brglez Mojzer E., Knez Hrnčič M., Škerget M., Knez Ž., Bren U. (2016). Polyphenols: Extraction Methods, Antioxidative Action, Bioavailability and Anticarcinogenic Effects. Molecules.

[78] Chepel V., Lisun V., Skrypnik L. (2020). Changes in the Content of Some Groups of Phenolic Compounds and Biological Activity of Extracts of Various Parts of Heather (*Calluna vulgaris* (L.) Hull) at Different Growth Stages. Plants.

[79] Vagiri M., Conner S., Stewart D., Andersson S.C., Verrall S., Johansson E., Rumpunen K. (2015). Phenolic Compounds in Blackcurrant (*Ribes nigrum* L.) Leaves Relative to Leaf Position and Harvest Date. Food Chem..

[80] Staszowska-Karkut M., Materska M. (2020). Phenolic Composition, Mineral Content, and Beneficial Bioactivities of Leaf Extracts from Black Currant (*Ribes nigrum* L.), Raspberry (*Rubus idaeus*), and Aronia (*Aronia melanocarpa*). Nutrients.

[81] Kuck L.S., Noreña C.P.Z. (2016). Microencapsulation of Grape (*Vitis Labrusca* Var. Bordo) Skin Phenolic Extract Using Gum Arabic, Polydextrose, and Partially Hydrolyzed Guar Gum as Encapsulating Agents. Food Chem..

[82] Tolun A., Altintas Z., Artik N. (2016). Microencapsulation of Grape Polyphenols Using Maltodextrin and Gum Arabic as Two Alternative Coating Materials: Development and Characterization. J. Biotechnol..

[83] Akdeniz B., Sumnu G., Sahin S. (2018). Microencapsulation of Phenolic Compounds Extracted from Onion (*Allium cepa*) Skin. J. Food Process. Preserv..

[84] Carra J.B., Matos R.L.N.D., Novelli A.P., Couto R.O.D., Yamashita F., Ribeiro M.A.D.S., Meurer E.C., Verri W.A., Casagrande R., Georgetti S.R. (2022). Spray-Drying of Casein/Pectin Bioconjugate Microcapsules Containing Grape (*Vitis labrusca*) by-Product Extract. Food Chem..

[85] Soleimanifar M., Jafari S.M., Assadpour E., Mirarab A. (2021). Electrosprayed Whey Protein Nanocarriers Containing Natural Phenolics; Thermal and Antioxidant Properties, Release Behavior and Stability. J. Food Eng..

[86] Csepregi R., Temesfői V., Das S., Alberti Á., Tóth C.A., Herczeg R., Papp N., Kőszegi T. (2020). Cytotoxic, Antimicrobial, Antioxidant Properties and Effects on Cell Migration of Phenolic Compounds of Selected Transylvanian Medicinal Plants. Antioxidants.

[87] Wang X., Liu X., Shi N., Zhang Z., Chen Y., Yan M., Li Y. (2023). Response Surface Methodology Optimization and HPLC-ESI-QTOF-MS/MS Analysis on Ultrasonic-Assisted Extraction of Phenolic Compounds from Okra (*Abelmoschus esculentus*) and Their Antioxidant Activity. Food Chem..

[88] Ciric A., Krajnc B., Heath D., Ogrinc N. (2020). Response Surface Methodology and Artificial Neural Network Approach for the Optimization of Ultrasound-Assisted Extraction of Polyphenols from Garlic. Food Chem. Toxicol..

[89] Kratchanova M., Denev P., Ciz M., Lojek A., Mihailov A. (2010). Evaluation of Antioxidant Activity of Medicinal Plants Containing Polyphenol Compounds. Comparison of Two Extraction Systems. Acta Biochim. Pol..

[90] Benamar-Aissa B., Gourine N., Ouinten M., Harrat M., Benarfa A., Yousfi M. (2023). Synergistic Effects of Essential Oils and Phenolic Extracts on Antioxidant Activities Responses Using Two Artemisia Species (A. campestris and A. herba alba) Combined with Citrus Aurantium. Biocatal. Agric. Biotechnol..

[91] Dif M.M., Benali Toumi F., Boukaaza H., Mokaddem F., Benyahia M., Bouazza S. (2018). Phenolic content and antioxidant activity of *Artemisa herba-alba*, a medicinal plant from Algerian arid zone. Phytothérapie.

[92] Bouchara N., Senejoux F., Fraisse D., Felgines C., Caldéfie-Chezet F., Vasson M.-P., Madani K., Rossary A. (2021). Anti-Inflammatory and Prolonged Protective Effects of *Artemisia herba-alba* Extracts via Glutathione Metabolism Reinforcement. S. Afr. J. Bot..

[93] Ayad N., Benaraba R., Hemida H., Abdellah F. (2022). Biological Activities of Phenolic Extracts from *Artemisia herba-alba* Asso Grown in Western Algeria. Eur. J. Biol. Res..

[94] Ghandehari Yazdi A.P., Barzegar M., Sahari M.A., Ahmadi Gavlighi H. (2021). Encapsulation of Pistachio Green Hull Phenolic Compounds by Spray Drying. J. Agric. Sci. Technol..

[95] Ferreira L.M.D.M.C., Pereira R.R., Carvalho-Guimarães F.B.D., Remígio M.S.D.N., Barbosa W.L.R., Ribeiro-Costa R.M., Silva-Júnior J.O.C. (2022). Microencapsulation by Spray Drying and Antioxidant Activity of Phenolic Compounds from Tucuma Coproduct (*Astrocaryum vulgare* Mart.) Almonds. Polymers.

[96] Akbarmehr A., Peighambardoust S.H., Soltanzadeh M., Jafari S.M., Sarabandi K. (2023). Microencapsulation of Yerba Mate Extract: The Efficacy of Polysaccharide/Protein Hydrocolloids on Physical, Microstructural, Functional, and Antioxidant Properties. Int. J. Biol. Macromol..

[97] Ghomari O., Sounni F., Massaoudi Y., Ghanam J., Drissi Kaitouni L.B., Merzouki M., Benlemlih M. (2019). Phenolic Profile (HPLC-UV) of Olive Leaves According to Extraction Procedure and Assessment of Antibacterial Activity. Biotechnol. Rep..

[98] Tsali A., Goula A.M. (2018). Valorization of Grape Pomace: Encapsulation and Storage Stability of Its Phenolic Extract. Powder Technol..

[99] Navarro-Flores M.J., Ventura-Canseco L.M.C., Meza-Gordillo R., Ayora-Talavera T.D.R., Abud-Archila M. (2020). Spray Drying Encapsulation of a Native Plant Extract Rich in Phenolic Compounds with Combinations of Maltodextrin and Non-Conventional Wall Materials. J. Food Sci. Technol..

[100] Medfai W., Oueslati I., Dumas E., Harzalli Z., Viton C., Mhamdi R., Gharsallaoui A. (2023). Physicochemical and Biological Characterization of Encapsulated Olive Leaf Extracts for Food Preservation. Antibiotics.

[101] Tu Q.B., Wang P.Y., Sheng S., Xu Y., Wang J.Z., You S., Zhu A.H., Wang J., Wu F.A. (2020). Microencapsulation and Antimicrobial Activity of Plant Essential Oil Against Ralstonia Solanacearum. Waste Biomass Valorization.

[102] Maqsoudlou A., Sadeghi Mahoonak A., Mohebodini H., Koushki V. (2020). Stability and Structural Properties of Bee Pollen Protein Hydrolysate Microencapsulated Using Maltodextrin and Whey Protein Concentrate. Heliyon.

[103] Cassol L., Noreña C.P.Z. (2021). Microencapsulation and Accelerated Stability Testing of Bioactive Compounds of Hibiscus Sabdariffa. J. Food Meas. Charact..

[104] Ćujić-Nikolić N., Stanisavljević N., Šavikin K., Kalušević A., Nedović V., Bigović D., Janković T. (2018). Application of Gum Arabic in the Production of Spray-Dried Chokeberry Polyphenols, Microparticles Characterisation and in Vitro Digestion Method. Lek. Sirovine.

[105] Fazaeli M., Emam-Djomeh Z., Kalbasi Ashtari A., Omid M. (2012). Effect of Spray Drying Conditions and Feed Composition on the Physical Properties of Black Mulberry Juice Powder. Food Bioprod. Process..

[106] Başyiğit B., Sağlam H., Kandemir Ş., Karaaslan A., Karaaslan M. (2020). Microencapsulation of Sour Cherry Oil by Spray Drying: Evaluation of Physical Morphology, Thermal Properties, Storage Stability, and Antimicrobial Activity. Powder Technol..

[107] Baghi F., Ghnimi S., Dumas E., Gharsallaoui A. (2023). Microencapsulation of Antimicrobial Trans-Cinnamaldehyde: Effect of Emulsifier Type, pH, and Drying Technique. Appl. Sci..

[108] Tonon R.V., Brabet C., Hubinger M.D. (2008). Influence of Process Conditions on the Physicochemical Properties of Açai (*Euterpe oleraceae* Mart.) Powder Produced by Spray Drying. J. Food Eng..

[109] Daza L.D., Fujita A., Fávaro-Trindade C.S., Rodrigues-Ract J.N., Granato D., Genovese M.I. (2016). Effect of Spray Drying Conditions on the Physical Properties of Cagaita (*Eugenia dysenterica* DC.) Fruit Extracts. Food Bioprod. Process..

[110] Quek S.Y., Chok N.K., Swedlund P. (2007). The Physicochemical Properties of Spray-Dried Watermelon Powders. Chem. Eng. Process. Process Intensif..

[111] Hoyos-Leyva J.D., Bello-Perez L.A., Agama-Acevedo J.E., Alvarez-Ramirez J., Jaramillo-Echeverry L.M. (2019). Characterization of Spray Drying Microencapsulation of Almond Oil into Taro Starch Spherical Aggregates. LWT—Food Sci. Technol..

[112] Ocak B. (2020). Gum Arabic and Collagen Hydrolysate Extracted from Hide Fleshing Wastes as Novel Wall Materials for Microencapsulation of *Origanum onites* L. Essential Oil through Complex Coacervation. Environ. Sci. Pollut. Res..

[113] Goula A.M., Adamopoulos K.G. (2010). A New Technique for Spray Drying Orange Juice Concentrate. Innov. Food Sci. Emerg. Technol..

[114] Botrel D.A., de Barros Fernandes R.V., Borges S.V., Yoshida M.I. (2014). Influence of Wall Matrix Systems on the Properties of Spray-Dried Microparticles Containing Fish Oil. Food Res. Int..

[115] Feihrmann A.C., Da Silva N.M., De Marins A.R., Antônio Matiucci M., Nunes K.C., Nakamura C.V., De Souza M.L.R., De Oliveira O., Gomes R.G. (2024). Ultrasound-Assisted Extraction and Encapsulation by Spray Drying of Bioactive Compounds from Tradescantia Zebrina Leaves. Food Chem. Adv..

[116] Joye I.J., McClements D.J. (2014). Biopolymer-Based Nanoparticles and Microparticles: Fabrication, Characterization, and Application. Curr. Opin. Colloid Interface Sci..

[117] Sarabandi K., Peighambardoust S.H., Mahoonak A.S., Samaei S.P. (2017). Effect of Carrier Types and Compositions on the Production Yield, Microstructure and Physical Characteristics of Spray Dried Sour Cherry Juice Concentrate. J. Food Meas. Charact..

[118] Medina-Torres L., Santiago-Adame R., Calderas F., Gallegos-Infante J.A., González-Laredo R.F., Rocha-Guzmán N.E., Núñez-Ramírez D.M., Bernad-Bernad M.J., Manero O. (2016). Microencapsulation by Spray Drying of Laurel Infusions (*Litsea glaucescens*) with Maltodextrin. Ind. Crops Prod..

[119] Ballesteros L.F., Ramirez M.J., Orrego C.E., Teixeira J.A., Mussatto S.I. (2017). Encapsulation of Antioxidant Phenolic Compounds Extracted from Spent Coffee Grounds by Freeze-Drying and Spray-Drying Using Different Coating Materials. Food Chem..

[120] Do Nascimento T.G., Borges A.L.T.F., De Almeida L.M., Ribeiro Ê.A.N., Silva F.G.C., Da Costa Silva V., Do Nascimento Prata A.P., Basílio-Júnior I.D., Goulart M.O.F., Morilla D.P. (2022). Preparation and Characterization of Microcapsules Loaded with Polyphenols-Enriched Uncaria Tomentosa Extract Using Spray-Dryer Technique. J. Therm. Anal. Calorim..

[121] De Abreu Figueiredo J., Andrade Teixeira M., Henrique Campelo P., Maria Teixeira Lago A., Pereira De Souza T., Irene Yoshida M., Rodrigues De Oliveira C., Paula Aparecida Pereira A., Maria Pastore G., Aparecido Sanches E. (2020). Encapsulation of Camu-Camu Extracts Using Prebiotic Biopolymers: Controlled Release of Bioactive Compounds and Effect on Their Physicochemical and Thermal Properties. Food Res. Int..

[122] Estupiñan-Amaya M., Fuenmayor C.A., López-Córdoba A. (2020). New Freeze-Dried Andean Blueberry Juice Powders for Potential Application as Functional Food Ingredients: Effect of Maltodextrin on Bioactive and Morphological Features. Molecules.

[123] Tirgarian B., Farmani J., Farahmandfar R., Milani J.M., Van Bockstaele F. (2023). Switchable pH-Responsive Biopolymeric Stabilizers Made by Sonothermal Glycation of Sodium Caseinate with Κappa-Carrageenan. Food Biophys..

[124] Arrieta M.P., Peltzer M.A., Garrigós M.D.C., Jiménez A. (2013). Structure and Mechanical Properties of Sodium and Calcium Caseinate Edible Active Films with Carvacrol. J. Food Eng..

[125] Otálora M.C., Wilches-Torres A., Gómez Castaño J.A. (2023). Microencapsulation of Betaxanthin Pigments from Pitahaya (*Hylocereus megalanthus*) By-Products: Characterization, Food Application, Stability, and In Vitro Gastrointestinal Digestion. Foods.

[126] Xue R., Yuan X., Jiang H., Huang H., Luo X., Li P. (2024). Preparation and Physicochemical Analysis of Camellia Sinensis Cv. ‘Ziyan’ Anthocyanin Microcapsules. Foods.

[127] Rigolon T.C.B., Silva R.R.A., De Oliveira T.V., Nascimento A.L.A.A., De Barros F.A.R., Martins E., Campelo P.H., Stringheta P.C. (2024). Exploring Anthocyanins-Polysaccharide Synergies in Microcapsule Wall Materials via Spray Drying: Interaction Characterization and Evaluation of Particle Stability. Meas. Food.

[128] Bisinella R.Z.B., De Oliveira C.S., Zappani P.S.C., Schnitzler E., Masson M.L. (2016). Thermal Analysis as Screening Technique to Assess Spray-Drying Process of Encapsulated “Yacon” Juice. J. Therm. Anal. Calorim..

[129] Nunes G.L., Boaventura B.C.B., Pinto S.S., Verruck S., Murakami F.S., Prudêncio E.S., De Mello Castanho Amboni R.D. (2015). Microencapsulation of Freeze Concentrated Ilex Paraguariensis Extract by Spray Drying. J. Food Eng..

[130] Castro-López C., Espinoza-González C., Ramos-González R., Boone-Villa V.D., Aguilar-González M.A., Martínez-Ávila G.C.G., Aguilar C.N., Ventura-Sobrevilla J.M. (2021). Spray-Drying Encapsulation of Microwave-Assisted Extracted Polyphenols from *Moringa oleifera*: Influence of Tragacanth, Locust Bean, and Carboxymethyl-Cellulose Formulations. Food Res. Int..

[131] Zahnit W., Smara O., Bechki L., Bensouici C., Messaoudi M., Benchikha N., Larkem I., Awuchi C.G., Sawicka B., Simal-Gandara J. (2022). Phytochemical Profiling, Mineral Elements, and Biological Activities of *Artemisia campestris* L. Grown in Algeria. Horticulturae.

[132] Khenifi M.L., Serseg T., Migas P., Krauze-Baranowska M., Özdemir S., Bensouici C., Alghonaim M.I., Al-Khafaji K., Alsalamah S.A., Boudjeniba M. (2023). HPLC-DAD-MS Characterization, Antioxidant Activity, α-Amylase Inhibition, Molecular Docking, and ADMET of Flavonoids from Fenugreek Seeds. Molecules.

[133] Benouchenne D., Bellil I., Akkal S., Bensouici C., Khelifi D. (2020). LC–MS/MS Analysis, Antioxidant and Antibacterial Activities of Algerian Fir (*Abies numidica* de LANNOY Ex CARRIÈRE) Ethylacetate Fraction Extracted from Needles. J. King Saud Univ.—Sci..

[134] Djermane N., Gali L., Arhab R., Gherraf N., Bensouici C., Erenler R., Gok M., Abdessamed A. (2020). Chemical Composition and in Vitro Evaluation of Antioxidant, Antimicrobial, and Enzyme Inhibitory Activities of Erucaria Uncata and Thymeleae Hirsuta. Biocatal. Agric. Biotechnol..

[135] Bendjedid S., Lekmine S., Tadjine A., Djelloul R., Bensouici C. (2021). Analysis of Phytochemical Constituents, Antibacterial, Antioxidant, Photoprotective Activities and Cytotoxic Effect of Leaves Extracts and Fractions of Aloe Vera. Biocatal. Agric. Biotechnol..

[136] Guebebia S., Gharsallaoui A., Dumas E., Baghi F., Zourgui L., Romdhane M., Agusti G., Ghnimi S. (2023). Microencapsulation of Phenolic Compounds Extracted from Okra (*Abelmoschus esculentus* L.) Leaves, Fruits and Seeds. Appl. Sci..

